# Two-Stage Hybrid Approach of Deep Learning Networks for Interstitial Lung Disease Classification

**DOI:** 10.1155/2022/7340902

**Published:** 2022-02-01

**Authors:** Swati P. Pawar, Sanjay N. Talbar

**Affiliations:** ^1^SVERI's College of Engineering Pandharpur, India; ^2^Center of Excellence in Signal and Image Processing, SGGS Nanded, India

## Abstract

High-resolution computed tomography (HRCT) images in interstitial lung disease (ILD) screening can help improve healthcare quality. However, most of the earlier ILD classification work involves time-consuming manual identification of the region of interest (ROI) from the lung HRCT image before applying the deep learning classification algorithm. This paper has developed a two-stage hybrid approach of deep learning networks for ILD classification. A conditional generative adversarial network (c-GAN) has segmented the lung part from the HRCT images at the first stage. The c-GAN with multiscale feature extraction module has been used for accurate lung segmentation from the HRCT images with lung abnormalities. At the second stage, a pretrained ResNet50 has been used to extract the features from the segmented lung image for classification into six ILD classes using the support vector machine classifier. The proposed two-stage algorithm takes a whole HRCT as input eliminating the need for extracting the ROI and classifies the given HRCT image into an ILD class. The performance of the proposed two-stage deep learning network-based ILD classifier has improved considerably due to the stage-wise improvement of deep learning algorithm performance.

## 1. Introduction

As the risk of lung cancer incidences among patients with interstitial lung disease (ILD) is high [1], identifying the specific type of ILD becomes essential to develop appropriate therapy plans. In the healthcare industry, data-driven decision-making [2] is becoming popular due to its ability to quickly gather and analyze complete and accurate data. It makes the decision-makers choose an appropriate treatment, predict future events, and plan long-term action. ILD classification problems can be linked with diagnosing a particular type of ILD, forecasting the spread of ILD and further implementing preventive measures.

The HRCT image-based processes are the preliminary screening approach for the quick visualization of normal and abnormal cases of any disease. In addition, the data-driven decision-making approach for ILD classification can become effective for the early detection of ILD. The image-based classification approach involves feature extraction and labeling the ILD class to train the classifier. Feature extraction involves efficient shape, texture, and colour extraction for spatial and frequency-based image analysis. These methods include gray level values [3], texture feature extraction, statistic filters such as gray level cooccurrence matrix and run length [4], edge features such as Gaussian and Wavelet filters [5], and spatial and shape features [6]. However, these features will not capture the features of deep learning proposed by deep learning algorithms.

With the advancement in deep learning algorithms, feature extraction in medical image analysis has become more reliable. These algorithms are used for solving various applications in the domain of detection [7], segmentation [8], and classification [9]. Deep learning algorithms such as AlexNet [10], VGG [11], and GoogLeNet [12] help to obtain deep feature vectors. However, this architecture needs a massive amount of data for training and testing, which becomes difficult in medical fields and is sometimes tedious and time-consuming. This data scarcity issue, which may result in overfitting, is resolved by transfer learning [13], which inherits or preserves the knowledge learned from a data-rich source domain. These features are labeled after appropriate feature extraction to implement the machine learning algorithms. The most frequently used supervised classification algorithms are support vector machine [4], *K*-nearest neighbor [14], Bayesian classifiers [15], linear discriminant analysis [16], and artificial neural network [3]. The most promising classifier for giving the great value of true positive rates and accuracy is SVM. A detailed review of deep learning approaches for solving various medical imaging problems has been covered by Santosh et al. [17].

Most ILD classification works are based on the regions of interest (ROI) patch-based image representation. Li et al. [18] designed a CNN with a shallow convolution layer to classify ILD automatically and efficiently to learn the intrinsic image features from lung image patches that are most suitable for the classification purpose. Song et al. [19] proposed a new locality-constrained subcluster representation ensemble model to classify HRCT images of ILDs. It helps in the separation between different classes for improving classification performance. Anthimopoulos et al. [20] proposed and evaluated Leaky ReLU activation function-based CNN for classification of ILD. The ILD of seven patterns shows a classification performance of about 85.5%. Doddavarapu et al. [21] proposed architecture for automatic ILD classification using CNN with three convolution layers, Leaky ReLU activation followed by maximum pooling layer and dense layer. The last fully connected layer has five outputs equivalent to five ILDs, which gives an accuracy of about 94%. Guo et al. [22] developed an improved DenseNet called small kernel DenseNet to improve ILD classification performance and show the significant performance improvement compared to earlier CNNs AlexNet, VGGNet, and ResNet.

A different approach based on whole X-ray images was demonstrated by Poap et al. [23] and Sahlol et al. [24] to detect lung diseases and TB diagnostics. Further, Khan et al. [25] proposed a framework to support automated segmentation and classification of lung nodules with improved accuracy using VGG-SegNet for nodule mining and pretrained DL-based classification to help automatic lung nodule detection. Finally, the approach of using whole HRCT images for ILD classification was proposed by Gao et al. [26], which brought out drawbacks of image patch-based methods. Though the technique had advantages for handling large-scale image processing and analysis, the success rate was comparatively low. The low success rate was due to unwanted noise introduced by the background of HRCT images. The present study explores a multistage deep learning network to improve the ILD classification performance. Earlier, this approach was demonstrated by Elsayed et al. [27] for emotion recognition in Arabic news headlines and by Zhong and Gu [28] for capturing complex malware data distribution.

The literature survey shows the following significant gaps that can be addressed through the proposed two-stage hybrid approach-based classifier: (a) use of ROI patch, which is time-consuming and needs manual expert intervention, (b) the small HRCT patches may not fully capture the visual details and spatial context, (c) the use of traditional image processing algorithms for lung nodule segmentation, and (d) most of the classifiers developed in the literature use directly ROI patches instead of developing the classifier right from the HRCT images. The highlights of the proposed two-stage hybrid approach for ILD classification have been given below:

- The proposed algorithm uses whole HRCT images to eliminate human expertise requirement for manual extraction of ROI of ILD-infected part of the lung
- In the two-stage hybrid approach, at the first stage, a conditional generative adversarial network (c-GAN) with a multiscale feature extraction module has been used for accurate lung segmentation from the HRCT images with lung abnormalities. The lung segmentation removes the unwanted background from HRCT images, helping the next stage deep learning algorithm focus on the lung's ILD features
- The ResNet50 has been used to extract the deep features from the segmented lung images in the second stage. In addition, the pretrained ResNet50 has been fine-tuned based on the transfer learning approach using the segmented lung images of different ILD classes
- Finally, the support vector machine (SVM) utilized the deep features from ResNet50 to classify the six ILD classes, viz., normal, emphysema, fibrosis, ground glass, micronodules, and consolidation
- Overall, ILD classification performance gets improved due to selection c-GAN, which is suitable for lung segmentation and ResNet50, which is ideal for deep feature extraction. Also, improving the accuracy of deep learning algorithms at each stage will improve the overall performance
- The performance of the proposed algorithm has been compared with earlier patch-based image input and whole image input algorithms

The paper has been organized in the following sections. The significance of the proposed two-stage hybrid approach for ILD classification is brought out through the literature survey in Section 1.  Section 2 describes a two-stage hybrid approach of deep learning networks for ILD classification giving the details of deep learning algorithms used at each stage. The experiments that demonstrate the proposed two-stage hybrid approach and its performance analysis have been discussed in Section 3. The advantages and limitations of the proposed approach are discussed in Section 4. Finally, the conclusions of the proposed work have been given in Section 5.

## 2. Architecture of Two-Stage Hybrid Approach of Deep Learning Networks

**Figure 1:**
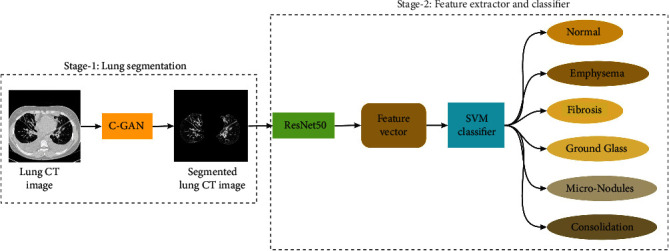
Architecture of the two-stage hybrid approach.

Most of the existing ILD classifiers involve manual identification of regions of interest (ROI) as a prerequisite to screen potential disease. Further, the patches of ROIs have been given as input to the deep learning algorithms for mapping the ILD classes. In this study, the two-stage architecture of a deep learning algorithm has been proposed, which connects two separate deep learning algorithms utilized for deep feature extraction and ILD classification.  Figure 1 shows the two-stage hybrid approach of deep learning networks for ILD classification, which takes the whole HRCT images as input and gives the ILD class label as output. The first stage deep learning algorithm segments the lung part from given HRCT images by removing background noise. Therefore, the segmented lung HRCT images from the first stage have been given as input to the second stage, where the features have been extracted using another deep learning algorithm. Further, in the second stage, the memory-efficient classifier support vector machine (SVM) has been used to classify ILDs based on the features obtained by the second stage deep learning algorithm. The following paragraphs have given brief descriptions of the deep learning networks used in the proposed algorithm.

### 2.1. Stage 1 c-GAN for Lung Segmentation

**Figure 2:**
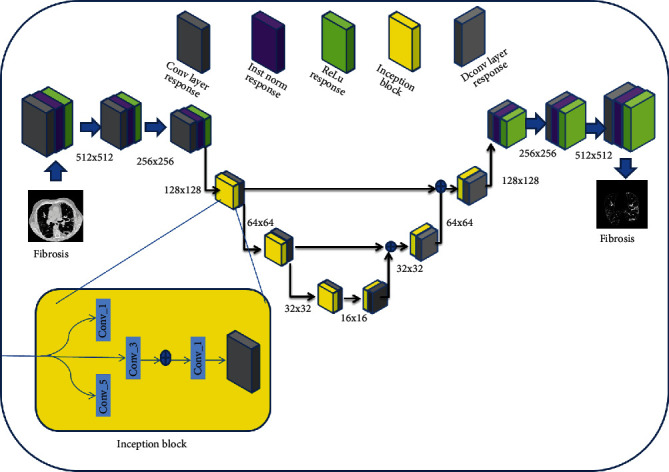
Conditional generative adversarial network (c-GAN) used for lung segmentation at stage 1.

Conditional generative adversarial network (c-GAN) is advantageous to use for the segmentation purpose due to its two main subcomponents, viz., generator (*G*) and discriminator (*D*) [29]. The role of the generator is to generate fake images using latent samples. At first, the generator generates the images using random pixels. Further, the generator has been trained to map these random variables to recognizable images, which can fool the discriminator (*D*). The generator (*G*) maps the input lung HRCT slice (*x*) with the reference lung segmentation map (*y*) as *G* : {*x*, *z*}⟶*y*, whereas the discriminator (*D*) discriminates between the generator output and the reference lung segmentation map.  Figure 2 shows the architecture of the proposed c-GAN, which consists of encoder/decoder blocks and multiscale feature extraction (MSFE) module. The encoder/decoder blocks have been formed by convolution/deconvolution filters of size 3 × 3, followed by the ReLU nonlinear action function. These blocks encode the input lung HRCT slices into feature maps that have been further normalized using the instance normalization [30] approach. The feature maps have been further downsampled in the encoder blocks for increasing the receptive fields. The upsampling of these features has been performed in the decoder blocks by the factor of 2 for maintaining the symmetry of the network.

The role of MSFE is to extract features which take care of dense abnormalities in the lung HRCT scans of different sizes, shapes, and textures. Most of the existing lung segmentation algorithms fail to include these dense abnormalities present, especially at the lung border. The inclusion of MSFE in the proposed algorithm captures the feature due to variation in the appearance of abnormalities. The MSFE includes inception blocks in which the input feature maps pass through three convolution layers of the filter size, i.e., 1 × 1, 3 × 3, and 5 × 5, followed by the ReLU and instance normalization. As shown in Figure 2, the proposed c-GAN segmentation architecture consists of six MSFE modules. The first three MSFE modules process the multiscale features through a simple convolution layer with a stride factor of 2. The remaining three MSFE modules maintain symmetry by processing these features through a simple deconvolution layer with the upsampling factor of 2. Thus, the MSFE module architecture helps network uniform appearance (i.e., size, shape, and texture) of the dense abnormalities on lung CT slice by capturing the prominent edge information in the output lung segmentation map.

The training of the proposed c-GAN network has been performed by calculating the losses of the generator and discriminator. The discriminator loss is the sum of losses of the real and fake images. The generator and discriminator variables have been updated separately.

The proposed c-GAN network has been trained by solving the objective function:

(1) $$\begin{array}{c} G^{*}=\arg\underset{G}{\text{m}}\text{in}\underset{D}{\text{m}}\text{ax}L\left(G,D\right), \end{array}$$

using Adam's stochastic optimization approach. The overall loss function is given by

(2) $$\begin{array}{c} L\left(G,D\right)=\ell_{\text{cGAN}}\left(G,D\right)+\lambda \cdot \ell_{L1}\left(G\right). \end{array}$$

The multiobjective probabilistic function of conditional GAN *ℓ*_cGAN_(*G*, *D*) and the traditional loss *ℓ*_*L*_1__(*G*) can be expressed as [26]

(3) $$\begin{array}{c} \ell_{\text{cGAN}}\left(G,D\right)=E_{x,y}\left[\log D\left(x,y\right)\right]+E_{x,\text{z}}\left[\log\left(1-D\left(x,G\left(x,z\right)\right)\right)\right],\\ \ell_{L_{1}}\left(G\right)=\underset{i,j}{\sum }{\left\|G{\left(x,z\right)}_{i,j}-y_{i,j}\right\|}_{2}. \end{array}$$

The training is aimed at training the discriminator to maximize the probability of the training data and to minimize the probability of the data sampled from the generator. Simultaneously, the generator has been trained on the opposite objectives as maximizing the probability that the discriminator is assigning to its samples.

### 2.2. Stage 2 ResNet50 for Feature Extraction and SVM for Classification

As shown in Figure 1, the segmented lung image received from stage 1 has passed through ResNet50 for deep feature extraction. ResNet50 is a pertained CNN based on feature transmission to prevent gradient vanishing, such that a much deeper network than those used previously could be effectively trained [31]. Based on the principle that the deeper network is more powerful than a shallow network, ResNet50 includes a 50-layer residual network architecture with 177 layers. The ResNet50 has been pretrained on a subset of the ImageNet database (http://www.image-net.org), and the architecture details are shown in Table 1.

Like any other deep network, the ResNet50 network consists of all the components like convolution, pooling, activation, and fully connected layers stacked one over the others. The only differentiator that makes it a residual network is that the identity connection between the layers. The identity connections resolve the problem of vanishing gradient problem. The residual blocks get skipped at once, and gradients will reach the initial layers, which will help to learn the correct weights. In ResNet50, the ReLU function is placed after the addition operation, which helps in changing the gradient values as they enter inside the residual block. As shown in Table 1, the ResNet50 architecture has four levels which can take input images of 224 × 224 × 3. The network performs the initial convolution and max pooling with kernel sizes of 7 × 7 and 3 × 3, respectively.

Further, level 1 has three residual blocks containing three layers, each performing convolution operations at all three layers. These three layers are 1 × 1, 3 × 3, and 1 × 1 convolutions to form bottleneck design. Thus, there are three identity connections between the three blocks of level 1. The convolution operation between level 1 and level 2 has been performed with stride 2, which doubles the channel width, whereas the size of inputs has been reduced to half. Similarly, for the next two levels, while progressing from one level to another level, the channel width has been doubled, and sizes of inputs have reduced to half as height and width.

The deep features obtained from a particular layer of the ResNet50 are fed to SVM classifier for ILD classification. The network extracts various classification-related deep features in each layer and pass to the next layer. The activation is in GPU with a minibatch size of 64, and GPU memory has enough space to fit the image dataset. The output from the activation function has been used to fit in SVM training. The SVM [32] used here is based on the function “fit class error-correcting output codes (fitcecoc),” which returns the fully trained multiclass error-correcting output of the model. The “fitcecoc” uses binary SVM models with One-Vs-All, and error-correcting output coding design enhances the performance of classification models. As the proposed algorithm utilized several deep features obtained from ResNet50, the SVM classifier is a suitable option due to its memory-efficient approach of handling high-dimensional spaces.

## 3. Experiments

Performance analysis of the proposed ILD classifier has been demonstrated using an ILD database prepared by Depeursinge et al. [33]. This database includes the lung HRCT slices with annotations that have been prepared by a discussion with the radiologists, research physicians, and computer scientists during the four years of the project period. The selected ILD database provides a common platform for evaluating automated ILD analysis systems, which 108 HRCT scans with annotated lung field maps. The images of six classes considered in the study have been taken as normal, emphysema, ground glass, fibrosis, micronodules, and consolidation for the analysis. The training dataset has been prepared by taking the ILD dataset out of 108 HRCT scans, whereas the remaining dataset has been used to validate the proposed optimal lung segmentation network. The network was trained using about 4000 HRCT slices created by the data augmentation approach, which considers the *flip left*, *flip right*, *flip up-down*, *image transpose* operators to increase the training dataset size. Other parameters of the model are similar to [21].

### 3.1. Lung Segmentation Performance

Lung segmentation performance of stage 1 c-GAN network has been obtained in the form of dice similarity coefficient (DSC) and Jaccard index (*J*), which has been given as

(4) $$\begin{array}{c} \text{DSC}=\frac{2\;|\;G\left(x\right)\cap y\;|\;}{\;|\;G\left(x\right)\;|\;+\;|\;y\;|\;},\\ J=\frac{\text{DSC}}{2-\text{DSC}}. \end{array}$$

Usually, the lung segmentation performance deteriorates in the presence of ILD due to the dense abnormalities present in the lung HRCT images of different sizes, shapes, and textures.  Table 2 shows the average lung segmentation performance analysis of the c-GAN carried out on 22 lung CT scans from the ILD database, compared with the existing state-of-the-art deep networks, *viz*., *NMF* [34], UNet [35], ResNet [31], VGG16 [10], and MobileNet [36]. The tables show that the performance of c-GAN and other existing networks depends on the ILD present in the HRCT image. The c-GAN method used in this work outperforms other existing methods for lung segmentation. The performance analysis shows that the c-GAN network gives near-perfect lung segmentation than the ground truth. In contrast, other methods fail to include lung abnormalities accurately.

### 3.2. ILD Classification Performance

**Figure 3:**
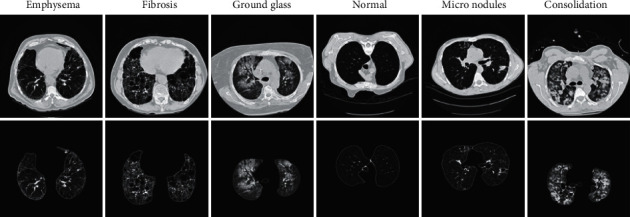
Examples of HRCT of six ILD. (The first row shows original HRCT images and the second row shows respective segmented image.)

In the second stage, the segmented images labeled with six ILD classes as normal, emphysema, fibrosis, ground glass, micronodules, and consolidation have been used to extract the deep features using pretrained ResNet50 mapping to the ILD classes.  Figure 3 shows representative images of original HRCT and segmented form for all the six ILD classes. The overfitting on image recognition training has been minimized by increasing the number of images in the database by augmentation. In addition, it has been ensured that the labels of the augmented images are being preserved. During the augmentation process, the CT images of 512 × 512 × 3 have been resized to 224 × 224 × 3. These images have been passed to pretrained ResNet50 for extracting the deep features. At this stage, appropriate feature layer selection becomes essential for improving the performance of the classifier. In the pretrained network, the weights of earlier layers are frozen, which were not updated during the fine-tuning stage. Therefore, the transfer learning techniques used for ResNet50 become advantageous for such problems as it removes the drawback of the shortage of radiological images required to improve deep learning performance. The transfer learning process effectively develops classification algorithms with less training data of medical images.

Before the detailed performance analysis of the classifier, the influence of feature layer selection on the classifier accuracy has been studied. Out of the trials conducted using the selection of feature layers as fcc1000, res5c_branch2b, res5c_branch2c, avg_pool, and bn5c_branch2a, SVM classifier gives the best result for the deep features selected at bn5c_branch2a.

The classification performance is measured using the performance parameters recall, precision, *F*-score, and accuracy given below:

(5) $$\begin{array}{c} \text{Recall}=\frac{\text{TP}}{\text{TP}+\text{FN}},\\ \text{Precision}=\frac{\text{TP}}{\text{TP}+\text{FP}},\\ F\text{score}=\frac{2\text{TP}}{2\text{TP}+\text{FP}+\text{FN}},\\ \text{Accuracy}=\frac{\text{TP}+\text{TN}}{\text{TP}+\text{TN}+\text{FP}+\text{FN}}, \end{array}$$

where TP is the number of true positives for the classification of ILD. Similarly, FP is the false positives, TN is the true negatives, and FN is the false negatives.

Statistical consistency of the proposed algorithm has been analyzed using 30 different cycles of randomly selected groups of HRCT images. The confusion matrices of the classifier obtained for the 30 cycles are presented in a 95% confidence interval (CI) in Table 3. This table shows the mean values and their variations for 95% confidence interval for the six ILD classes considered in this study. The table shows that the normal class, which is healthy, gives the highest accuracy of 94.65% (with 95% CI variation of 93.06%-96.24%) and gets slightly mixed with by about 2.55% with ground glass and micronodule disease. On the other hand, the lowest accuracy is for the consolidation, 84.12% (with 95% CI variation of 85.41%-82.83%), which gets confused with fibrosis and ground glass. However, the classification performance of the proposed classifier for the consolidation class has been considerably improved due to the segmented image inputs compared to the whole image input classifier [26], which was just 46.67%.

**Figure 4:**
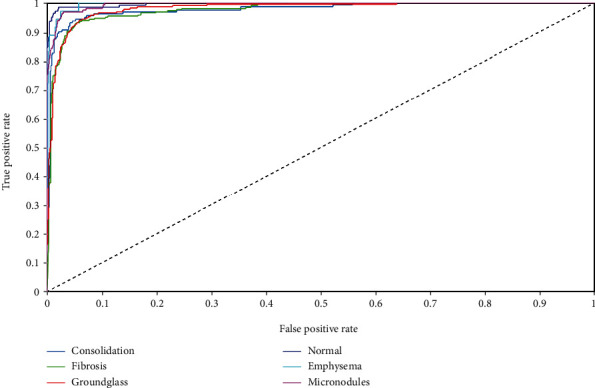
ROC plots of SVM classifier for the six ILD classes.

Further, Table 4 shows the interaction of six ILD classes for the proposed classifier obtained as precision, recall, and *F*-score values from the mean values of confusion matrix of Table 3. The average values of the precision, recall, and *F*-score are 89.65%, 89.39%, and 89.45%, respectively.  Figure 4 shows the ROC curves for the six classes, and their AUC values are given in Table 4. The ROC plots and AUC values indicate that the classifier is well trained, and classification results are obtained with a sufficient classification margin.

Table 5 compares the performance of the proposed two-stage hybrid ILD classifier with the earlier works in the literature. Most of the methods have used lung image patches of size 32 × 32, and only one method proposed by Gao et al. [26] used the whole images for ILD classification. Most of the earlier patch-based ILD classifiers have considered only five classes and have not included the consolidation class. Most of the existing patch-based ILD classifiers give average values of *F*-score, and accuracy for five classes is less than about 78%. Recently developed patch-based classier developed by Doddavarapu et al. [21] provides average values of *F*-score, and accuracy for five classes is about 94%. The proposed classifier shows nearly matching performance by giving the accuracy of about 90% to classify six classes. Thus, the proposed classifier performs better even with six ILD classes. A similar work of developing an ILD classifier using whole HRCT images to classify six ILD classes was developed by Gao et al. [26]. The average values of *F*-score and accuracy for this classifier are 66.83% and 69.23%, far lower than those of the proposed classifier. The proposed algorithm's accuracy is improved due to automatic preprocessing of unwanted noisy parts of HRCT images by the lung segmentation process performed at stage 1.

### 3.3. Implementation of the ILD Classification Process

A PC with 4.20 GHz Intel Core i7 processor and NVIDIA GTX 1080 8 GB GPU was used to train the two-stage hybrid approach of deep learning networks for ILD classification. For the segmentation training using around 4000 images, the c-GAN network, which is a shallow network, took about 2000 seconds. On the other hand, it takes approximately 19.20 seconds for the training of ResNet50 for deep feature extractions using around 1000 images, and SVM training took around 17.85 seconds. During testing of the two-stage hybrid approach, the total time required per image was about 0.7 seconds which is the sum of about 0.5 seconds taken for stage 1 and about 0.2 seconds taken for stage 2. Therefore, the time requirement analysis indicates that the two-stage hybrid approach of ILD classifier did not take much additional computational time compared to giving the advantage of improved classification accuracy.

## 4. Discussion

The proposed ILD classification approach is based on utilizing the whole HRCT images. Though this approach has the advantage of eliminating the dependency on human expertise required for manual extraction of ROI of ILD, it will not be able to mark the specific region infected by ILD. The time needed to scan many HRCT images using the ROI approach will be high. However, the proposed approach will monitor the whole image to classify it into a particular ILD class. The proposed ILD classifier has been developed by assuming that the given input HRCT image has only one type of ILD. However, the ILD classifier needs to be improved for handling the input images with multiple ILDs, which should bring out all the classes or severe classes of the given image. Another limitation of the proposed algorithm is that it is helpful to classify only five significant ILDs and will not work for several other ILDs which are not considered in this study.

## 5. Conclusions

A two-stage hybrid approach of deep learning networks has been proposed to screen interstitial lung disease (ILD) using whole HRCT images. Improving the accuracy of deep learning algorithms at each stage has improved the classifier's overall performance. At the first stage, the lung segmentation removes the unwanted background from HRCT images, helping the next stage to accurately extract ILD features from the lung image. Deep features from the segmented lung images have been extracted using ResNet50. The multiclass support vector machine algorithm utilizes the deep learning features to classify into six ILD classes, viz., normal, emphysema, fibrosis, ground glass, micronodules, and consolidation. The performance of the proposed algorithm has been compared with earlier patch-based and whole image-based algorithms. The highest classification accuracy of 94.65% has been obtained for the healthy class, which helps in reducing the chances of false alarm situations. The lowest classification accuracy of 84.12% has been obtained for the consolidation class, which is far better than other whole image-based algorithms. The proposed algorithm, which has considered six ILD classes, performs considerably better than existing algorithms with five classes and gives far better performance than a similar whole image-based algorithm. The proposed approach brings out the potential of improving the overall performance by choosing the appropriate CNN for a given task and improving accuracies at each stage of the functions. Furthermore, the additional time required for the proposed multistage CNN is negligible.

## Figures and Tables

**Table 1:** Details of the ResNet50 network for extracting deep features at stage 2.

Layer name	Optimal size	Sublayer
CONV1	112 × 112	7 × 7, 64, stride 2
CONV2_x	56 × 56	3 × 3 max pool stride 2 $\left[\begin{array}{c} 1\times 1,64\\ 3\times 3,64\\ 1\times 1,128 \end{array}\right]\times 3$
CONV3_x	28 × 28	$\left[\begin{array}{c} 1\times 1,128\\ 3\times 3,128\\ 1\times 1,512 \end{array}\right]\times 4$
CONV4_x	14 × 14	$\left[\begin{array}{c} 1\times 1,256\\ 3\times 3,256\\ 1\times 1,1024 \end{array}\right]\times 6$
CONV5_x	7 × 7	$\left[\begin{array}{c} 1\times 1,512\\ 3\times 3,512\\ 1\times 1,2048 \end{array}\right]\times 3$
	1 × 1	Average pool, 1000-d fc, softmax
FLOPs		3.8 × 10^9^

**Table 2:** Comparative performance assessment of average DSC and *J* for c-GAN and existing methods for lung segmentation.

Disease	Performance	Present study	NMF [34]	UNet [35]	ResNet [31]	VGG16 [10]	MobileNet [36]
Fibrosis	DSC	0.9566	0.7681	0.9485	0.9126	0.9295	0.9040
	*J*	0.9290	0.6600	0.9117	0.8681	0.8742	0.8330

Ground glass	DSC	0.9558	0.8335	0.9534	0.9351	0.9444	0.9291
	*J*	0.9282	0.7473	0.9191	0.8987	0.8975	0.8706

Emphysema	DSC	0.9378	0.9214	0.9629	0.9261	0.9452	0.9380
	*J*	0.9204	0.8917	0.9340	0.8975	0.8963	0.8841

Consolidation	DSC	0.9712	0.8775	0.9500	0.9440	0.9479	0.9436
	*J*	0.9466	0.7963	0.9148	0.9076	0.9031	0.8954

Micronodule	DSC	0.9812	0.9678	0.9807	0.9674	0.9751	0.9586
	*J*	0.9645	0.9391	0.9627	0.9379	0.9523	0.9210

**Table 3:** Confusion matrix of the proposed classifier (mean and variation values with 95% confidence interval).

Actual cases	Prediction (%)					
	Emphysema	Fibrosis	Ground glass	Normal	Micronodules	Consolidation
Emphysema	93.24 ± 1.66	5.56 ± 1.36	0.37 ± 0.34	0.00	0.37 ± 0.34	0.46 ± 0.46
Fibrosis	0.55 ± 0.23	89.26 ± 1.54	4.43 ± 0.91	0.22 ± 0.14	0.77 ± 0.46	4.76 ± 0.77
Ground glass	0.28 ± 0.26	5.27 ± 1.37	84.44 ± 2.17	4.70 ± 0.87	2.23 ± 0.84	3.08 ± 0.66
Normal	0.05 ± 0.06	0.16 ± 0.13	2.57 ± 1.14	94.65 ± 1.59	2.55 ± .70	0.02 ± 0.05
Micronodules	0.02 ± 0.04	0.26 ± 0.19	4.56 ± 0.73	3.45 ± 0.85	90.64 ± 1.42	1.06 ± 0.41
Consolidation	0.10 ± 0.08	9.84 ± 1.21	4.67 ± 0.67	0.21 ± 0.19	1.07 ± 0.43	84.12 ± 1.29

**Table 4:** ILD classifier interactive performance analysis of the proposed algorithm.

	Emphysema	Fibrosis	Ground glass	Normal	Micronodules	Consolidation	Avg
Precision (%)	98.94	80.89	83.57	91.68	92.84	89.96	89.65
Recall (%)	93.24	89.26	84.44	94.65	90.64	84.12	89.39
*F*-score (%)	96.00	84.87	84.00	93.14	91.73	86.94	89.45
AUC	0.9960	0.9769	0.9811	0.9969	0.9948	0.9793	0.9875

**Table 5:** Comparison of the proposed classifier with the earlier CNN-based classifiers.

Method	Image input type	*F*-score (%)	Accuracy (%)
Li et al. [17]	ROI patch	66.57	67.05
LeNet [37]	ROI patch	67.83	67.90
AlexNet [38]	ROI patch	70.31	71.04
Pretrained AlexNet [38]	ROI patch	75.82	76.09
VGGNet [10]	ROI patch	78.04	78.00
Doddavarapu et al. [21]	ROI patch	94.65	94.67
Gao et al. [26]	Whole HRCT	66.83	69.23
Proposed two-stage hybrid classifier	*Segmented HRCT*	**89.45**	**89.39**

## Data Availability

No data were used to support this study.
